# Exploring the impact educational interventions have on nursing and medical students’ attitudes and empathy levels towards people with disability. A systematic review

**DOI:** 10.1177/17446295231155781

**Published:** 2023-02-16

**Authors:** William Evans, Dominika Lisiecka, Dawn Farrell

**Affiliations:** 8813 Department of Nursing and Healthcare Sciences, School of Health and Social Sciences, Munster Technology University Tralee, Ireland; 8813 Munster Technology University Kerry, Tralee, Ireland; 8813 Munster Technology University Kerry, Tralee, Ireland

**Keywords:** Systematic review, Disability, Empathy, Attitudes, Education

## Abstract

This systematic review aimed to explore the impact educational interventions have on undergraduate nursing and medical students' attitudes and empathy levels towards people with disability. There are over one billion people with some form of disability currently. A growing body of research reveals that nurses and doctors display negative attitudes including decreased empathy towards people with disability. A systematic review using narrative synthesis of chosen randomized controlled trials was employed. A comprehensive search was completed in June 2021 on six databases (CINAHL, Medline, Science Direct, Health Research Premium - PROQUEST, Scopus. Cochrane Library). The search strategy yielded 21,616 studies and only three randomised controlled trials fulfilled the eligibility criteria. These trials included 125 participants (*n* = 50 medical students and *n* = 75 nursing students) and evaluated the effectiveness of a disabled health course, disability education module with bedside teaching and wheelchair workshop intervention. Findings from one study revealed that a disabled health course using affective learning method based on a transformative learning theory significantly improves attitudes to disability amongst nursing students however there was no statistically significant difference in empathy levels. More high-quality randomised controlled trials with greater theoretical and methodological complexity are needed to identify more effective educational approaches that enhance attitude and empathy levels of these key stakeholders.

## Introduction

Approximately one billion people have some form of disability and this figure is expected to grow further as a result of demographic shifts in populations as well as an increase in chronic conditions (World Health Organisation, 2022). This suggests that nurses and doctors as members of the wider healthcare team are having increased contact and exposure to people with disabilities. Providing high standards of care and support to people with disabilities (Iezzoni et al., 2021) free of discrimination is a prerequisite to how health care workers perform their duties (Sağlık, 2020). The language within medical and nursing regulatory guidance is unambiguous in relation to professional practice and discrimination; for example the Nursing and Midwifery Board of Ireland (NMBI) state in their Code of Professional Conduct and Ethics: ‘You must respect all people equally and not discriminate on grounds of (…) disability’ (NMBI, 2021: p. 11). Equally the General Medical Council in the UK state: ‘Never discriminate unfairly against patients or colleagues’ (General Medical Council, 2015: pg.2). However negative attitudes towards people with disability permeate amongst health care workers (Iezzoni et al., 2021; Lyon and Houser, 2018; Polikandrioti et al., 2020). This can also manifest in family members of individuals with neurodevelopmental disorders, for example, having unpleasant experiences with healthcare professionals (Ceglio et al., 2020).

Attitudes to disability corresponds to behaviour (Oliva Ruiz et al., 2020; Sanbonmatsu et al., 2005) and they provide a lens as to how decisions are made by healthcare professionals (Chen and McNamara, 2020). A contemporary example of how attitude and behaviour to disability interlink emerged recently in regard COVID-19 and healthcare resource allocations. The pandemic ignited ‘complex ethical and legal questions’ arising from healthcare professionals having to reflect upon and consider whether or not people with a disability should be in the ‘best chance to recover’ category (Chen and McNamara, 2020: pg.511). Such ethical debate is not new within healthcare; for example, Ryan (2022), with specific reference to disability, has outlined health care responses by countries arising from limited organ transplantation and renal dialysis availability.

Another important attribute of care for people with disabilities is the expression of empathy (Jones and Miller, 2018). Empathy ’involves not only understanding the patient’s situation and feelings but also being able to communicate that understanding’ (Cecchetti et al., 2021: pg. 2). Evidence suggests that standards of empathy for vulnerable patients is not high within healthcare settings (Levett-Jones et al., 2017).

Nurses and doctors constitute the largest clinical staff groupings within healthcare (Lo et al., 2017; Ika et al., 2019). It is acknowledged in the empirical literature that negative attitudes amongst healthcare staff can have a direct impact on the quality of the interaction with people with a disability (Uysal et al., 2014; Med et al., 2013). Staff in these settings require appropriate knowledge levels in autism, for example, and a ‘supportive attitude to facilitate communication specific to this population’s needs’ (Corden et al., 2022: p. 387). In addition, studies have explored the impact of educational interventions on disability empathy levels of medical (Lynch et al., 2019) and nursing students (Levett-Jones et al., 2017).

However, to date, no research has synthesised existing evidence using a systematic review methodology. Arising from the growing literature in this area, the aim of this review is to explore the impact educational interventions have on nursing and medical students’ attitudes and empathy towards people with disability. This review defines disability as a substantial restriction in the capacity of the person to carry on a profession, business or occupation in the State or to participate in social or cultural life in the State by reason of an enduring physical, sensory, mental health or intellectual impairment (The Disability Act, 2005).

## Review Methods

### Aim

This systematic review aimed to explore the impact educational interventions have on undergraduate nursing and medical students' attitudes and empathy levels towards people with disability.

### Design

A systematic review (SR) using narrative synthesis.

### Search Methods

This review is reported in accordance with PRISMA guidelines (Page et al., 2021). The study protocol was registered in PROSPERO (CRD42021256829). The search strategy was developed and piloted in May 2021 with the assistance from the University Librarian. Electronic systematic literature searches were performed in June 2021 using the following databases: CINAHL, Medline, ScienceDirect, Health Research Premium - PROQUEST, Scopus, and Cochrane Library. The keyword search concepts included: disability, education, attitudes, nursing and medical students. A comprehensive range of synonyms for each key search concepts were devised and used in the systematic search. Medical Subject Headings framework along with Boolean terms, wildcards and proximity indicators were applied as per each database configuration. The searches were limited to peer-reviewed studies in English published within the last ten years (2011-2021). An overview of the search strategy is located in Table 1.

**Table 1.** Search Strategy Overview.

**Review Topic**: Exploring the impact educational interventions have on nursing and medical students’ attitudes and empathy towards people with disability. **Research Question**: For nursing and medical undergraduate students, what impact do specified educational interventions have on their attitudes towards people with disability? **Eligibility criteria**: 1. Randomised Controlled Trials (RCT) will be sought, and in the absence of RCTs, quasi-experimental studies will be chosen. Single arm studies will only be chosen in circumstances where there is an absence of quasi-experimental studies. This is a pragmatic approach influenced by the Joanna Briggs Institute (JBI Manual for Evidence Synthesis, Chapter 3. Conducting systematic reviews of effectiveness, 2020). 2. English language. 3. Human studies. 4. Peer-reviewed studies published in full (not solely as conference abstracts). 5. 10 year time restriction will be applied. 6. Studies which recruited undergraduate nursing and medical students only (studying as part of their primary studies/degree). Qualified nurses or doctors studying part-time or full time will not the eligible. Postgraduate students will not be eligible.	
**Search**: Six databases were systematically searched: CINAHL, Medline, Science Direct, Health Research Premium - PROQUEST, Scopus. Cochrane Library. Medical Subject Headings (MeSH) framework along with Boolean, wildcards and proximity indicators were applied as per the database configuration. The keyword search terms included a variety of synonyms which describe: disability, education, attitudes, nursing and medical students (see below). Owing to time restriction length, synonym searching will include disability descriptions that include those that are outdated and discriminatory. Appropriate and relevant references cited in included studies will also be hand-searched.	
**Date:** 23/06/2021	
**Key search concept**	**Synonyms**
**Disability**	Disability OR ‘intellectual disability’ OR ‘physical disability’ OR ‘developmental delay’ OR ‘developmental disability’ OR ‘disabled people’ OR ‘disabled person’ OR ‘learning disability’ OR ‘learning difficulty’ OR impairment OR retard OR handicap.
**Education**	Educat* OR learning OR training OR pedagogy OR support OR curriculum OR module OR course, OR ‘skills session’ OR ‘knowledge acquisition’ OR ‘experiential placement visit’ OR presentation OR tutorial OR workshop OR ‘Educational intervention’ OR ‘educational program*’
**Nurse and medical Student**	(Nurs* OR medical OR doctor) AND (student OR undergraduate OR trainee OR assistant).

The search results were imported into the web application Endnote initially (for references management and re-duplication purposes) and then into Rayyan (Ouzzani et al., 2016) for further reduplication and to manage the screening process. Three authors performed independent reviews of titles and abstracts. Following this initial screening process, the three authors then read all articles identified for full text review to determine if the studies met the inclusion criteria. Studies were discussed until a consensus was reached. The reference lists of all review articles on the topic of interest identified during the search and all articles included in the full text review were also hand-searched to identify any eligible studies which may have been omitted in the electronic search. Study screening commenced in June and was finalised in August 2021. A pragmatic approach influenced by the Joanna Briggs Institute (Aromataris and Munn, 2020) was employed; randomised control trials (RCTs) were sought, and in the absence of RCTs, quasi-experimental studies were to be chosen.

## Search Outcome

Studies were eligible if the intervention had a specific purpose to educate nursing or medical students about physical and/or intellectual disabilities either as a distinct educational program or as part of their educational curriculum. An educational intervention included the provision of information on disabilities including such topics as social justice, human rights, inclusion, supporting autonomy, models of disability and advocacy, for example. The educational intervention could have been delivered across a large and diverse number and blend of modalities including but not limited to written, face-to face-classes, online, experiential, skill based and onsite. Attitudes to disability was the primary outcome and empathy and adverse events were the secondary outcomes.

### Quality Appraisal

The methodological quality of included studies was evaluated independently by two authors using the Cochrane Collaboration’s tool for assessing risk of bias in randomised trials (Higgins et al., 2019) before a consensus meeting took place with all authors. Where data were absent or lacking in clarity, the corresponding author was contacted for further information by email with two follow-up reminders.

### Data Extraction

For those articles meeting the eligibility criteria, two authors independently completed data extraction using the standardised ‘Data collection form for intervention reviews: RCTs only’ (The Cochrane Developmental Psychosocial and Learning Problems Review Group, 2014). Each study was analysed and a data abstraction table was completed.

### Data Analysis/Synthesis

Individual trials were assessed based on the differences and similarities in the interventions, participant groups and outcomes. It was determined by consensus and agreement was reached that the included trials were heterogeneous in nature and pooling of studies was not possible. Therefore, a narrative approach to analysis of the results of individual trials was conducted.

## Results

### Search Results

**Figure 1.**
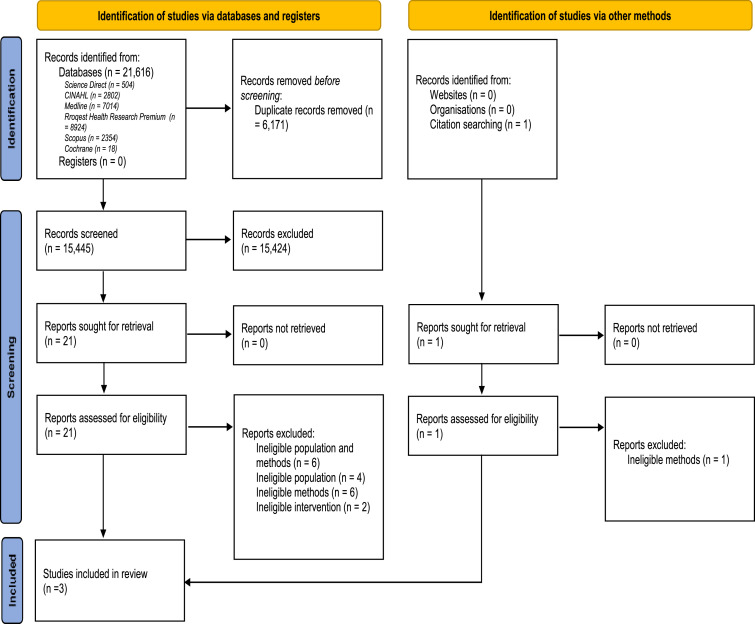
PRISMA Flow Diagram.

The electronic search identified a total of 21,616 citations of which 15,445 were screened by title and abstract following reduplication (see Figure 1: PRISMA flow diagram). The search of conference abstracts and reference lists yielded one further potentially eligible citation. The full texts of 22 studies were examined in detail. A total of three trials were identified as meeting the inclusion criteria and were included in this review (Dincer and Inangil, 2021; Santoro et al., 2019; Kirby et al., 2011).

### Study Characteristics and Quality

All studies were RCTs, including one cluster randomised controlled trial (Santoro et al., 2019). One of the studies did not provide any educational intervention to the control group (Kirby et al., 2011) while the other two studies used conventional educational approaches described as classroom based (Dincer and Inangil, 2021) and didactic lectures (Santoro et al., 2019).

All three trials were completed unblinded (Dincer and Inangil, 2021; Kirby et al., 2011; Santoro et al., 2019). The method of group allocation was not reported in any trial. Outcomes were assessed before and after the intervention in two trials (Santoro et al., 2019; Dincer and Inangil, 2021), however in one trial data were only collected at six days post the intervention (Kirby et al., 2011).

Two trials were conducted on medical (n=50) (Kirby et al., 2011; Santoro et al., 2019) and one on nursing students (n=75) (Dincer and Inangil, 2021). Sample size ranged from 24 participants (Santoro et al., 2019) to 75 participants (Dincer and Inangil, 2021), therefore most sample sizes were small. Two trials were undertaken in single universities (Kirby et al., 2011; Dincer and Inangil, 2021), one trial involved a university paediatric hospital and county teaching hospital setting (Santoro et al., 2019). Participants’ age ranged from a mean of 20.44 years (Dincer and Inangil, 2021) to aged ≥25 in 78% of the participants (Santoro et al., 2019). Participants were predominantly female. Baseline characteristics of the groups were not reported in one trial (Dincer and Inangil, 2021), with no imbalance reported by Kirby et al. (2011), but there was a statistically significant difference in baseline characteristics in terms of personal experience with disability and ethnicity in one trial (Santoro et al., 2019). Trials used different inclusion criteria: type of students and year of study only (Santoro et al., 2019) and also age, availability and volunteer criteria (Kirby et al., 2011; Dincer and Inangil, 2021). One trial excluded students who had attended any course for disabled persons previously or lived with a disabled family member (Dincer and Inangil, 2021). Loss to follow up ranged from no loss (Santoro et al., 2019) to 8% (n=2) of the sample (Kirby et al., 2011) and it was not reported in one trial (Dincer and Inangil, 2021).

In terms of interventions, two trials were general disability educational programmes, including a disability education module with bedside teaching (Santoro et al., 2019) and a disabled health course (Dincer and Inangil, 2021). One trial focused specifically on a wheelchair education intervention (Kirby et al., 2011). Two of the interventions had a theoretical basis: transformative learning theory (Dincer and Inangil, 2021) and Gardner’s Theory (Kirby et al., 2011). Transformative learning theory requires learners to become conscious of their attitudes, to have these directly challenged, to reflect personally and communally on these challenges and to integrate learning into a new perspective’ (Thompson et al., 2016: p.868). Kirby et al., (2011) used Gardner’s theory which sets out to acknowledge the varied and diverse learning styles of participants (Kirby et al., 2011). In addition, the theory assists in the ‘personalisation of the training’ and the exclusion of ‘uniform’ and ‘inefficient teaching’ (Oprescu and Craciun, 2011: , pg.87)

All interventions were delivered as face-to-face group sessions. The duration of the intervention included a one-time four hour workshop (Kirby et al., 2011), two lectures and two bedside teaching sessions facilitated over 2 weeks (Santoro et al., 2019) to a 14-week course of 2-hour lessons per week (Dincer and Inangil, 2021).

Attitudes to disability were assessed in all three trials using different scales. Dincer and Inangil (2021) used the Attitudes toward Disabled Persons scale (Yuker et al., 1970), Kirby et al. (2011) used the Scale of Attitudes toward Disabled Persons (Antonak, 1982), and Santoro et al (2019) used a self-developed measure of attitudes toward child disability. The secondary outcomes of this review, empathy and adverse events, each were assessed in one trial. Empathy was assessed by Dincer and Inangil (2021) using the Empathic Tendency Scale (Dökmen, 1988) and adverse events were qualitatively self-reported by Kirby et al. (2011).

All three studies were randomised. Studies used sequence generation methods such as randomisation tables (Kirby et al., 2011), computer based random number generator (Dincer and Inangil, 2021) or random assignment for their core rotations to one of two clinical sites (Santoro et al., 2019). Concealment of allocation was unclear due to insufficient information reported. With regard to performance bias, all three studies were unblinded due to the inherent nature of the interventions. Two of the trials assessed outcomes via self-reports therefore contributing to high risk of detection bias as unblinded studies (Dincer and Inangil, 2021; Kirby et al., 2011) and one trial indicated that each pre- and post-clerkship survey was reviewed and scored independently by two authors, who were blinded to the group during scoring phase (Santoro et al., 2019). Surveys were assigned post hoc to the appropriate group based on coded identifier (Santoro et al., 2019). There was no dropout (Santoro et al., 2019) or a relatively low dropout rate (Kirby et al., 2011) yielding a low risk of bias classification for incomplete outcome data. Dincer & Inangil (2021) did not report loss to follow up. Data were not reported in standardised summary format (sample mean and standard deviation), with findings for the primary outcome in this review reported as medians (Dincer and Inangil, 2021), percentage scores (Kirby et al., 2011) or thematic in nature (Santoro et al., 2019). Therefore, all three trials were judged as having high risk of bias for selective outcome reporting. One trial reported similar baseline characteristics between intervention and control groups (Kirby et al., 2011), however, unbalanced groups at baseline were noted in one trial (Santoro et al., 2019), so this study was rated as having a high risk of selection bias. No information on baseline characteristics were reported by Dincer and Inangil (2021), therefore it was rated as unclear for selection bias. One trial used a valid scale to measure attitudes to disability (Dincer and Inangil, 2021), however two trials (Kirby et al., 2011; Santoro et al., 2019) did not report the validity of the scales used. Therefore, the reliability of the study findings needs to be interpreted with caution.

**Figure 2.**
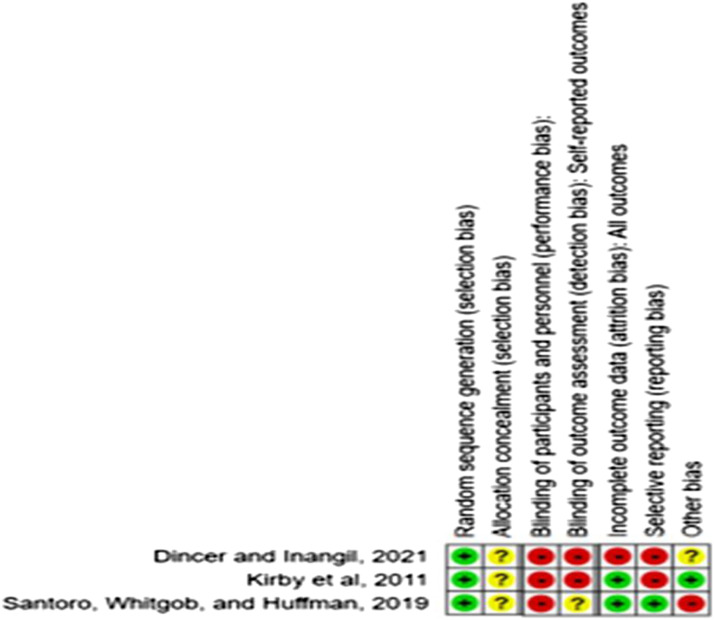
Risk of Bias Analysis.

None of the three trials were judged as adequately meeting all criteria on the risk of bias tool (See Figure 2). Further characteristic of trials and details on the risk of bias for each individual trial are located within the 'Characteristics of included studies' (See Table 2).

**Table 2.** Characteristics of Included Studies.

Dincer and Inangil, 2021		
**Methods**	Randomized controlled trial	
**Participants**	Nursing students studying at degree level in a Department of Nursing Inclusion criteria: (a) being 18 years old or over, (b) studying in the second year (c) volunteering to take a disability health course (d) not attending any course for disabled persons before (e) not living with a disabled family member. A total of 75 nursing students were randomised and allocated to the intervention (n = 34) or control group (n = 36). Age [Mean (SD) years]: intervention group 20.44 (0.78); control group not reported Gender: intervention group (88.2% females, 11.8% males); control group (91.7% females, 8.3% males) Race/ethnicity not reported Loss to follow up: not reported	
**Interventions**	Disabled health course using affective learning method, based on transformative learning theory Mode of delivery: a) face to face; delivered by two nurse educators experienced in the field of disability Duration: 14 weeks, 2 hour lesson per week Setting if trial: Single Department of Nursing in Istanbul, Turkey. Setting of intervention: Classroom in the Department of Nursing Comparison treatment: Disabled health course delivered using traditional learning method. Co-interventions: Not reported	
**Outcomes**	Attitudes to Disability was assessed using the Attitudes Toward Disabled Person Scale Empathy was assessed using the Empathic Tendency Scale Adverse events were not assessed Assessment time points: Before and after disabled health course	
**Notes**	Additional information supplied by the author	
**Risk of Bias**		
**Bias**	Authors Judgement	Support for Judgement
**Random sequence generation (selection bias)**	Low risk	A randomly ordered list was issued according to the student numbers In the classroom list by using the website random.org. A computer based random number generator was used to divide students into groups. An independent statistician carried out the randomization.
**Allocation concealment** **(selection bias)**	Unclear	No information on method of allocation reported
**Blinding of participants** **and personnel (performance bias)** **All outcomes**	High risk	Unblinded RCT
**Blinding of outcome assessment (detection bias)** **All outcomes**	High risk	Self-reported measures and unblinded RCT
**Incomplete outcome data** **(attrition bias)** **All outcomes**	High risk	Loss to follow up was not reported. Reasons for exclusion detailed but loss following allocation not reported
**Selective reporting (reporting bias)**	High risk	Results of outcome reported as medians - mean and standard deviation data not reported
**Other bias**	Unclear	Baseline characteristics of the groups not reported

Santoro, Whitgob, and Huffman, 2019		
**Methods**	Cluster randomized controlled trial	
**Participants**	24 medical students (second-sixth year of medical education) who were on their pediatrics core clerkship from May to October 2016 Inclusion criteria: Medical student year 2 – 6 A total of 12 (52%) students randomized to the intervention group and 11 (48%) students randomized to the control group. Age group (years) Aged ≤24 : Total n = 5 (22%); Control n = 3 (27%); Intervention n = 2 (17%); Aged ≥25: Total n = 18 (78%); n = 8 (73%); n = 10 (83%) Female Total n =12 (52%); Control n = 7 (64%); Intervention n = 5 (42%) Male Total n = 11 (48%); Control n = 4 (36%); Intervention n = 7 (58%) Race White Total n = 5 (22%); Control n = 2 (18%); Intervention n = 3 (25%) Black/African American Total n = 3 (13%); Control n = 1 (9%); Intervention n = 2 (17%) Asian Total n = 12 (52%); Control n = 8 (73%); Intervention n = 4 (33%) Other Total n = 3 (13%); Control n = 0 (0%); Intervention n = 3 (25%) Ethnicity Hispanic Total n = 7 (30%); Control n = 1 (9%); Intervention n = 6 (50%) Non-Hispanic Total n = 16 (70%); Control n = 10 (90%); Intervention n = 6 (50%) Training level MS2 Total n = 1 (4%); Control n = 0 (0%); Intervention n = 1 (8%) MS3 Total n = 18 (79%); Control n = 8 (73%); Intervention n = 10 (84%) MS4 Total n = 3 (13%); Control n = 3 (27%); Intervention n = 0 (0%) ≥MS5 Total n = 1 (4%); Control n = 0; Intervention n = 1 (8%) Personal experience with disability None Total n= 9 (39%); Control n = 7 (64%); Intervention n = 2 (17%) A little Total n = 11 (48%); Control n = 4 (36%); Intervention n = 7 (58%) A lot Total n = 3 (13%); Control n = 0 (0%); Intervention n = 3 (25%) Professional experience with disability None Total n = 10 (43%); Control n = 4 (36%); Intervention n = 6 (50%) A little Total n= 13 (57%); Control n = 7 (64%); Intervention n = 6 (50%) A lot Total n = 0 (0%); Control n = 0 (0%); Intervention n = 0 (0%) Loss to follow up: No loss to follow up	
**Interventions**	Intervention Group Training Experience: *a*. Didactic Lecture #1: slide presentation on disability terms, the ADA, and history of disability. *b*. Bedside Teaching #1: participated in performing a guided physical and neurologic examination and interview of hospitalised child with a disability (cognitive, language, or motor) and his or her family members. *c*. Didactic Lecture #2: Two weeks later, slide presentation on disability examination techniques, followed by discussion of 5 clinical scenarios concerning patients with disability. *d*. Bedside Teaching #2: participated in performing a guided physical and neurologic examination and interview of hospitalized child (different than from bedside teaching #1) with a disability (cognitive, language, or motor) and his or her family members. Mode of delivery: a) face to face; delivered by fellow-level physician from division of Developmental and Behavioural Paediatrics or division of Child Neurology Duration: Two weeks Setting of trial: Massachusetts General Hospital, USA. Setting of intervention: University Children’s hospital Comparison treatment: Control Group Training Experience: a. Didactic Lecture #1: A senior paediatrics resident- level physician provided a 30-minute slide presentation on disability terms, the ADA, and US history of disability. b. No disabilities-focused bedside teaching occurred after this lecture. Co-interventions: Not reported	
**Outcomes**	Attitudes to Disability was assessed using a self-developed measure on attitudes concerning child disability Empathy and adverse events were not assessed Assessment time points: Before and after clerkship	
**Risk of Bias**		
**Bias**	Authors Judgement	Support for Judgement
**Random sequence generation (selection bias)**	Low risk	Students were randomly assigned for their core rotations to 1 of 2 clinical sites by a blinded clerkship coordinator. Those assigned to the university children’s hospital were automatically enrolled in the intervention group, and those assigned to the county teaching hospital were automatically assigned to the control group.
**Allocation concealment** **(selection bias)**	Unclear	No information on method of allocation reported
**Blinding of participants** **and personnel (performance bias)**	High risk	Unblinded RCT
**All outcomes**		
**Blinding of outcome assessment (detection bias)** **All outcomes**	Unclear	Each pre- and post-clerkship survey was reviewed and scored independently by 2 scoring authors (JDS and EEW), to confirm accurate scoring. Both scorers were blinded to the group during scoring phase, and surveys were assigned post hoc to the appropriate group based on coded identifier (street the participant grew up on).
**Incomplete outcome data** **(attrition bias)** **All outcomes**	Low risk	No loss to follow up
**Selective reporting (reporting bias)**	Low risk	Results of outcome reported, however thematic in nature
**Other bias**	High risk	Baseline imbalance in terms of personal experience with disability and ethnicity

Kirby, Crawford, Smith, Thompson, and Sargeant, 2011		
**Methods**	Randomized controlled trial	
**Participants**	Twenty-six medical students (11 first and 15 second-year students) Inclusion criteria: 17 yrs or older, was a student enrolled in the first or second year of the undergraduate medical education program. Inclusion criteria: Students had access to a computer and the Internet, was willing to participate, was competent to provide informed consent, was able to follow instructions, was available to participate on the day selected for the educational experience, and did not have any unstable physical or mental conditions that might make participation dangerous or unpleasant. 26 participants were randomly allocated to the intervention (n = 13) and control group (n = 13) Mean (SD) Age: Intervention 23.5 (1.2) Control 25.5 (2.8), p = 0.061 Male n (%): Intervention group n = 3 (25%); Control group n = 7 (58.3%), p= 0.098 Race/ethnicity: not reported First-year students, n (%): Intervention group n = 6 (50%); Control group n = 3 (25%), p = 0.400 Previous wheelchair use (yes), n (%): Intervention group n =1 (8.3%); Control group n = 3 (25%) p = 0.590 Relevant experience (yes) , n (%): Intervention group n = 8 (66.7%), Control group n = 7 (58.3) p = 1.000 Comfort level with disability, median: Intervention group : 4 ; control group : 4; p = 0.390 Loss to follow up (n = 2); intervention group (n = 1); control group (n = 1)	
**Interventions**	The wheelchair workshop included several learning activities, including didactic presentation, practical performance of wheelchair skills, a community experience, a reflective exercise, and self-study. Mode of delivery: face to face; delivered by an occupational therapist, a rehabilitation engineer, and a physiatrist instructors Duration: 4 hour educational workshop Setting of trial: Single University Setting of trial: Dalhousie University, Canada. Comparison treatment: Standard education Co-interventions: Not reported	
**Outcomes**	Attitudes to Disability was assessed using Scale of Attitudes Toward Disabled Persons (SADP) Empathy was not assessed Adverse event were self-reported Assessment time points: 6 days post workshop	
**Risk of Bias**		
**Bias**	Authors Judgement	Support for Judgement
**Random sequence generation (selection bias)**	Low risk	Participants were randomly allocated into two equal-sized groups (intervention and control), using a randomization table
**Allocation concealment** **(selection bias)**	Unclear	No information on method of allocation reported
**Blinding of participants** **and personnel (performance bias)** **All outcomes**	High risk	Unblinded RCT
**Blinding of outcome assessment (detection bias)** **All outcomes**	High risk	Self-reports and unblinded RCT
**Incomplete outcome data** **(attrition bias)** **All outcomes**	Low risk	Loss to follow up reported - One participant from each group dropped out because of scheduling difficulties.
**Selective reporting (reporting bias)**	High risk	Results of outcomes converted to percentage scores for analysis
**Other bias**	Low risk	There was no significant differences between the intervention and control groups with respect to baseline characteristics

### Findings

The summary of effects of the interventions on the predefined outcomes of the included trials are presented below.

#### Disabled health course using affective learning method versus traditional learning method

The effect of a disabled health course using affective learning method based on the transformative learning theory versus traditional learning method was assessed in one trial (Dincer and Inangil, 2021). The mean difference between the intervention and control groups could not be calculated for Attitudes Toward Disabled Person Scale (ATDP) and empathic tendency score, as summary data were published as medians and data were not available. However, based on median data, there was a statistically significant difference between the groups in terms of ATDP score after the disabled health course (p < 0.001). The median ATDP score after the intervention was 85.00 compared to 70.00 in the control group (p < 0.001). Also, there was a significant difference between the ATDP median scores before (Md = 69.00) and after (Md=85.00) in the intervention group (p < 0.001).

In contrast, there was no statistically significant difference between the groups in terms of empathic tendency score before (Md = 70.00) and after (Md = 70.00) the disabled health course (p = 0.063) (Dincer and Inangil, 2021).

#### Disability education module with bedside teaching versus no disabilities-focused bedside teaching

Attitudes towards disabilities as measured by a self-developed survey on attitudes concerning child disability was assessed in one trial (Santoro et al., 2019). Functional themes were formulated by two of the researchers following content analysis of the open-ended participant responses to questionnaire items. The content analysis of participants' post-intervention open-ended survey responses revealed that participants in the bedside teaching group used terminology in their responses that represented a functional understanding of disability with twice the frequency of the control group. The mean number of themes reflecting a functional understanding of disability reported by the intervention group was 3.75 compared to 2.0 in the control group. Two of these functional themes included: disparities in quality health care (p = 0.03) and care coordination (p < 0.001) and a statistically significant difference was found in both between the intervention and control groups.

#### Wheelchair workshop intervention versus standard education

Attitudes to disability were assessed using Scale of Attitudes Toward Disabled Persons (SADP) in one trial (Kirby et al., 2011). At six days post the workshop, there was no difference in mean SADP scores between the intervention and control groups. The mean SADP score was 126.45 in the intervention group and 128.75 in the control group (MD–2.30, 95% CI-12.15 to 7.55).

## Discussion

Educational interventions had an impact on attitudes to disability in only one of the three reviewed studies (Dincer and Inangil, 2021). Significantly, the findings from this review and the limited high-quality research in this area are concerning given that disability and human rights-based discourses echo strongly across the developed world. In ratifying the International Convention on the Rights of Persons with Disabilities, there is an expectation on countries to provide education to healthcare professionals on key competencies (Havercamp et al., 2021) including, for example, in the areas of human rights advocacy and autonomy.

The United Nations Convention on the Rights of Persons with Disabilities (UNCRPD (2007)) states that people with disability have a right to health care that is free from discrimination. The World Health Organisation (2022) concludes that many healthcare professionals ‘have limited knowledge and understanding of the rights of people with disability and their health needs and have inadequate training and professional development about disability’. All countries that have ratified the convention are responsible through both their legislature and policy structures to remove any outstanding barriers experienced by people with disabilities (Davies et al., 2019). Two of the studies included in this review were from countries (Turkey, Canada) that had previously ratified the UNCRPD (Dincer and Inangil, 2021; Kirby et al., 2011).

This review is the first to focus on this important area in disability discourse and following a considered and structured literature search and screening, three trials met the inclusion criteria. Given the global context of disability, it was expected that a large number of robust, methodologically strong RCTs would be completed in this area and available for inclusion for this review. Notwithstanding the small number of trials included in the review, and the overall low quality of evidence, committing to reporting the study in its entirety has advantages in that it provides a direction of travel for future research in this area. In addition, not completing a review based on there being limited or in some instances no studies to report, profits the notion of reporting bias (Gray, 2021).

The quality of evidence in this review is limited and there is a need to develop high quality RCTs with appropriate educational interventions that are fit for purpose to promote learning and enhance attitudinal change of nurses and doctors towards people with disability. A knowledge gap exists as to the nature and content of what constitutes ‘appropriate educational interventions’ with a lack of consensus as to what this means (Havercamp et al., 2021).

Two of the trials included in this review adopted a theoretical framework: Kirby et al., (2011) used Gardner’s theory (Gardner, 1983) discussed earlier in the review, and Dincer & Inangil, (2021) used transformative learning theory (Dincer and Inangil, 2021). Transformative learning theory situate the learner as a ‘reflexive intentional agents’ (Biesta, 2020: pg.55) and encourages students to question their ‘assumptions, beliefs, emotions, and perspectives’ in regards to disability (Dincer and Inangil, 2021: pg.4). Transformative learning is well recognised in the literature as an educational philosophy to enhance attitudinal change (Thompson et al., 2016) as well as in many other areas including inclusive education (Murdoch et al., 2020), health education (Van Schalkwyk et al., 2019), and in quality improvement and patient safety (Goldman et al., 2021). With a central focus on learning itself (Murdoch et al., 2020), the theory expedites perspective transformation and ensures that graduates develop knowledge, skills and competence in the cognitive, behavioural and emotional domains (Tsimane and Downing, 2020: pg.97). Learning with a focus on outcome, centres on what Stevens-Long et al. describe as ‘deep and lasting change, equivalent to what some people term a developmental shift or a change in worldview’ (Stevens-Long et al., 2011: pg.182).

Two main approaches in teaching disability to nursing and medical student were evident in this review including classroom and experiential, as well as a combination of both. The two studies that combined classroom and experiential approaches (Kirby et al., 2011; Santoro et al., 2019) did not achieve statistically significant change in attitudes to disability, however the study (Dincer and Inangil, 2021) that employed solely a classroom approach did achieve a desired outcome. Attitudes to disability therefore may be positively altered through classroom based educational interventions alone, however, further research is necessary to explore areas such as the theoretical underpinning and the role of transformative learning theory, given its strengths; the personnel necessary in the delivery; and programme duration. Furthermore, the three studies in this review did not include any online teaching, neither synchronous and asynchronous delivery, however the ‘new normality’ of post COVID-19 ‘has been the transformation to “digital” as a usable and functional educational platform’ (Haldar et al., 2022: p. 2).

Studies in this review that employed experiential approaches to teaching students about disability included bedside access (Santoro et al., 2019) and community experience (Kirby et al., 2011). Recent studies suggest that clinical encounters with people with disabilities can assist medical students become ‘more confident, comfortable, less awkward, and more skilled and efficacious’ (Crane et al., 2021: p.1). A recent systematic review explored the training requirements for medical professionals working with patients with neurodevelopment disability (Ceglio et al., 2020). A diversity of opportunities were identified to make clinical encounters with people with disabilities equally available to medical and nursing student cohorts using a variety of modified means, for example, home visits, community visits, school visits, shadowing, observation and in-clinic review (Ceglio et al., 2020). Edwards, Cron and Shonk (2022) further supported the inclusion of experiential learning focused on disability in the nursing curriculum to enhance confidence levels and skills when caring for individuals with disability, particularly as graduate nurses (Edwards et al., 2022).

Disability empathy levels of nursing and medical students were a secondary outcome of this review. One of the three included studies found no statistically significant difference in empathy levels between groups, following the transformative theory educational intervention (Dincer and Inangil, 2021). The findings are somewhat at odds with other studies in this area where evidence of growth in empathy levels were found by nursing (Levett-Jones et al., 2017; Geçkil et al., 2017) and medical students (Cecchetti et al., 2021; Lynch et al., 2019) following educational interventions. Kerasidou et al. argue that what makes ‘empathy possible’ for health care staff requires a broader ‘multi-tiered approach’ (Kerasidou et al., 2020: pg.4), which suggests that educational interventions alone cannot achieve an empathetic healthcare workforce. To make ‘empathy possible’ (Kerasidou et al., 2020), therefore requires discussion and meaningful action at regulatory, organisational and individual level.

An important question within this field of research centres on how educational interventions can sustain positive disability attitudinal and empathy levels amongst healthcare professionals such as doctors (Cecchetti et al., 2021) and nurses (Dincer and Inangil, 2021). The three trials in this review did not consider long term follow up and its effect on attitudes to disability. It is important that future research examines extended time periods post intervention such as 12 months, to assesses whether attitudinal change is retained more long term (Lynch et al., 2019). Another area requiring further exploratory research relates to disability attitudes and the impact prior experience and exposure plays. One trial in this review excluded those who attended a previous course for disabled persons or lived with a disabled family member (Dincer and Inangil, 2021), whereas the other two trials assessed these factors as study characteristics (Santoro et al., 2019; Kirby et al., 2011).

A final area requiring further investigation relates to disability attitudinal research and the role that gender plays. The sample characteristics of the three included trials identified that the participants were predominately female. The need to address genders more widely is needed considering Polikandrioti et al. (2020) identified that male student nurses had more positive attitudes to disability.

Positive developments are emerging in some countries with the development of core competencies in disability education for health care workers (Alliance for Disability, 2019). Developing a competency based educational framework aims to ensure that ‘learners have the capabilities necessary to provide high-quality care to patients with disabilities’ (Havercamp et al., 2021). However, as part of revising curriculum, a much deeper analysis is required to ensure that implicit and explicit disability biases associated with negative educational providers attitudes are not reinforced within curricula (VanPuymbrouck et al., 2020). Attitudinal barriers including stigmatization and discrimination when accessing health-related services are common experiences for people with disabilities (World Health Organisation, 2022). This review provides the necessary context to act as a lever for greater educational and research imagination so that nurses’ and doctors’ attitudes to people with disability will act as facilitators for positive change.

## Strengths and Limitations

This is the first review of its kind and is an important addition within the field of healthcare disability education. While the number of included trials was small, it provides the stimulus for future studies in this area. The review foregrounds important considerations for further investigations including the role of contemporary theoretical frameworks in educational interventions with the learner as centre, sustaining disability attitudinal change amongst healthcare students, and examining the role gender and past experiences play in this research field.

Limitations of this review include the limited number of trials with a small number of participants, overall low quality of evidence, sparse data, short follow up outcome assessment periods, and lack of blinding of intervention groups. Also, not all of the measures used in the trials reported psychometric properties; therefore, caution needs to be exercised when interpreting findings.

## Conclusions

Increasing demand for health and rehabilitation care amongst those with a disability (Uysal et al., 2014), coupled with an aging population profile (World Health Organisation, 2015) suggests that both doctors and nurses will have greater exposure with disability populations both now and into the future. Structured educational interventions for students have potential to positively influence professionals’ attitudes and empathy towards people with disability.

The limited number of RCTs examining the impact educational interventions have on disability attitudes and empathy levels of nursing and medical students presents ‘a call for action’ that future research is warranted in this area. Transformative learning offers a strong conceptual framework to guide future educational interventions that focus on the journey of learning as much as the outcome itself.

There is a need for considerably more robust, well-designed randomised controlled trials to identify effective interventions to improve attitudes and empathy of nursing and medical students towards individuals with disability. Future RCT’s in this area require extended follow-up outcome assessment period, larger sample sizes and more effective blinding processes to minimise potential biases. Disability competency frameworks operationalised by educational providers that do not harbour negative disability biases offer tangible solutions in this area. Finally, a case to widen the focus of a future systematic review to all healthcare professionals other than nursing and medical students should be considered.
